# Infantile Scurvy

**Published:** 1907-07

**Authors:** 


### INFANTILE SCURVY.

La Fetra observes that this disease has been recognized less than twenty years. It was formerly called acute rickets, but it was usually complicated with ulcerative stomatitis. Barlow gives the following as its chief features: 1. Predominance of lower limb involvement with (a) immobility, knee flexed, thigh rotated outward, pseudoparalysis, (b) excessive tenderness, (c) general swelling of the lower limbs due to subperiosteal hæmorrhage, (d) skin tense and shining, but seldom pitting and without local heat, (e) thickening of the shaft of the bone, (f) liability to fracture near the epiphysis. 2. Swelling of the gums ranging from sponginess to minute transient ecchymoses. 3. Tendency to hæmorrhage into the skin, subcutaneous tissues, mucous membranes, or viscera. 4. Definite and rapid improvement upon antiscorbutic diet. In treating this disease the cause must be removed. Devitalized food must be discontinued, and we must substitute fresh milk, fruit juice, beef juice, raw egg albumen, and puree of potato. Sterilized milk may be desirable under certain conditions, but better is fresh cows' milk which is produced under ideal hygienic conditions.—Amer. Jour. Med. Science, June, 1907.

